# ‘Terminal anorexia’: a lived experience perspective on the proposed criteria

**DOI:** 10.1186/s40337-023-00935-4

**Published:** 2023-12-11

**Authors:** Alykhan Asaria

**Affiliations:** London, UK

**Keywords:** Anorexia nervosa, Cognitive behavioural therapy, Compassion, Criteria, Eating disorder, Ego-syntonic, End-of-life, Hope, Identity, Label, Lived experience, Palliative, Terminal, Therapeutic alliance, Therapeutic relationship, Treatment-resistant

## Abstract

In an article (Asaria in J Eat Disord 11:107, 2023) recently published by the Journal of Eating Disorders, I expressed my lived experience views on the concept of ‘terminal anorexia nervosa’ (AN), and why I believe that this is a harmful new term. The article was not a response to the original paper in which Gaudiani et al. (J Eat Disord 10:23, 2022) proposed criteria for the label. However, as a result of feedback that my article did not appreciate their criteria, I have written this follow-up paper to build on and reinforce what I previously wrote. This article outlines problems with each criterion in turn, again from my lived experience perspective. It then addresses dangerous ambiguities around how the criteria can be applied safely, and their confusing purpose in the real world. Finally, I discuss the impact of labelling AN sufferers with terms that may suggest their wholehearted allegiance to the illness, in both life and death (or ‘till death do us part’).

## Brief summary of the proposed criteria

Gaudiani et al. [2] have proposed four criteria for the label ‘terminal AN’. They wrote in the abstract of their original article:

Consistent with literature on managing terminal illness, this article proposes clinical characteristics of patients who may be considered to have a terminal eating disorder:

1. Diagnosis of anorexia nervosa,
2. Older age (e.g., age over 30),
3. Previous participation in high quality care, and
4. Clear and consistent determination by a patient who possesses decision-making capacity that additional treatment would be futile, knowing their actions will result in death. [2, p. 1]

At the time of writing, these proposals have not changed. However, the authors recently wrote in Yager et al. [3]:

We clearly acknowledged that precise definition of the term remains to be developed and we invited the eating disorder and palliative care fields to systematically address these issues and develop consensus definitions and guidelines for these patients’ end-of-life care. [3, p. 2]

In the next four sections of this article, there are separate headings for each of the proposed criteria, with brief definitions requoted in the titles to serve as reminders. At the start of every section, a detailed description of the criterion in question is quoted.

## Criterion 1: “Diagnosis of anorexia nervosa” [2, p. 1]

Gaudiani et al. [2] proposed as criterion 1 in their original paper:

A diagnosis of anorexia nervosa. Anorexia nervosa is the only eating disorder that carries a guaranteed medical cause of death from malnutrition should weight loss continue unabated. As a result, consistent with literature on duration of life during hunger strikes resulting in death, a prognosis of less than 6 months can fairly be established when the patient acknowledges further treatment to be futile and stops engaging in active recovery work. A less than six-month prognosis is congruent with current practice around determination of terminal diagnoses. We fully recognize that patients with SEAN are likely to have other psychiatric conditions as well. [2, p. 11]

The 1st criterion makes it clear that only AN sufferers can be diagnosed as being ‘terminal’. Although it states that these individuals are likely to have comorbid psychiatric conditions, the criterion arguably does not *appreciate* this reality, as it implicitly assumes that those being considered will only die as a direct result of their AN. Furthermore, the 1st criterion does not acknowledge that coexisting conditions are often physical (e.g., diabetes, chronic fatigue syndrome) and/or neurodevelopmental [e.g., autism spectrum  disorder (ASD), attention deficit hyperactivity disorder (ADHD), learning difficulties].

From speaking to clinicians who know longstanding ED patients currently on ‘palliative pathways’, I understand that these individuals almost always have coexisting conditions. I have also read UK Court of Protection written judgements for nine extremely unwell AN sufferers considered between 2012 and the date of submission [4]. All of the nine judgements  have coexisting conditions *explicitly* recorded in them. These include: post-traumatic stress unrelated to past ED treatments (4 people); substance use disorders (4 people: 3 with alcohol dependency and 1 with opioid dependency); OCD (3 people); self-harm and suicidal behaviours (3 people); body dysmorphic disorder (2 people); major depressive disorder (2 people); personality disorders (2 people: 1 with emotionally unstable personality disorder and 1 with mixed personality disorder); bulimia nervosa (BN) (1 person);  ASD (1 person); and chronic fatigue syndrome with fibromyalgia (1 person).

All of the written judgements repeatedly mentioned unspecific/undiagnosed mental ill-health difficulties such as poor self-esteem, anxiety, panic attacks, low mood, mood instability, emotion dysregulation, trauma caused by past ED treatments (usually forced refeeding), binge/purge symptoms secondary to AN, and body image fears secondary to AN. They  usually indicated the presence of vulnerable personality traits (e.g., anxious/avoidant, dependent, obsessive–compulsive) and/or ASD traits.

I also came across a case involving an individual diagnosed with BN only (but without a diagnosis of AN). This shows that AN is not the only ED that can be life-threatening. BN patients can sometimes be at more risk due to the biological effects of purging, such as electrolyte imbalances, and high suicide rates caused by its associated impulsivity features [5]. However, the 1st criterion’s exclusive focus on AN reinforces an assumption that sufferers of other EDs do not deserve the same level of urgency when being cared for. This can lead to them feeling that they need to make themselves more physically ill in order to be taken seriously.

Concurrently, no sufferer of *any* ED needs to be labelled as ‘terminal’ when their physical symptoms are life-threatening. This does not happen for other mental illnesses that can be life-threatening due to the associated risks of suicide, self-harm (including accidental overdoses), self-neglect (including undereating caused by conditions like depression), and secondary physical health complications (including obesity-related conditions caused by atypical depression, liver damage caused by alcohol misuse, and lung cancer caused by higher rates of tobacco/cannabis smoking in schizophrenia sufferers). Wildgust et al. [6] reported that the life expectancy of schizophrenia patients is reduced by an average of 15–25 years, but young schizophrenia patients with terminal lung cancer are not unnecessarily diagnosed with ‘terminal schizophrenia’. As stated in my previous article:

Dying from the physical complications associated with a mental illness is not necessarily the same as dying from the mental illness itself (unless it is assumed that the whole person is the mental illness). [1, p. 4]

Regarding AN specifically, the effects of malnutrition are not guaranteed to be irreversible. In fact, the vast majority of them can be reversed [7, 8].^1^ On the other hand, ‘terminal AN’ is a label that is permanently tied to patients. The judgement may even be life-ending if it is wrongly interpreted as a certain and/or deserved death sentence. As stated in my previous article:

The possibility that AN can be terminal, even if it is caveated with messages that this is rare, is extremely disheartening to sufferers who are already losing hope. Their pessimistic belief systems and cognitive biases (especially labelling, all-or-nothing thinking, overgeneralising, selective attention, and arbitrary inference), which are often strongly reinforced by the way that they have been treated/discussed/labelled by clinicians for years, mean that many will assume they have death sentences and can never get better… Giving them a terminal diagnosis removes any hidden/silent hope that they may have. A sufferer who protests that there is no hope left may actually need the clinician to hold the hope for them, until they can carry it on their own. They may not feel deserving of the hope, or not know how to identify and express it. They may need clinicians to give them permission to live rather than permission to die. [1, p. 2 and p. 3]

## Criterion 2: “Older age (e.g., age over 30)” [2, p. 1]

Gaudiani et al. [2] proposed as criterion 2 in their original paper:

Age of 30 or older. This criterion accommodates for what is clinically seen as a potential ‘late maturation phase’ in which even those who have been sick for a long time may discover a shift in values and desires that motivates recovery as they enter their late 20s. Every effort should be made to promote full recovery and continuation of life in those younger than 30. However, the SMR [standardised mortality rate] data of multiple recent studies showing the highest death rates in those with a history of inpatient admissions, longer duration of AN, and age over 30 years old, taken alongside what functionally has often been a decade or two of exhaustive, ultimately unsuccessful eating disorder treatment, indicates that the age of around 30 as a minimum for terminal AN is reasonable. [2, p. 11]

The 2nd criterion sets an arbitrary and very low minimum age threshold of 30 for the classification of ‘terminal AN’. In doing so, Gaudiani et al. [2] have created their own definition of what *allows* an individual to be ‘young’. According to the 2nd criterion, sufferers are no longer young on their 30th birthday. In my view, as someone who just turned 30 a few days before submitting this article, it is unfair and demoralising to make such unevidenced judgements for any individual, let alone for AN sufferers who are likely to be more biologically, psychologically, and socially underdeveloped than their numerical age. The authors claim that the random number ‘30’ accommodates for a potential “late maturation phase”, but they provide no evidence to back up this assertion, which denigrates my own experiences.

Sufferers who are in their early 30s, *or* approaching this age, should not feel that they (will) suddenly no longer deserve what Gaudiani et al. refer to as “every effort… to promote full recovery and continuation of life in those younger than 30” [2, p. 11]. Guinhut et al. [9] found that the SMR of AN patients admitted to a specialised clinical-nutrition-unit (CNU) was highest (26.0) for 30–34 year-olds. Surely, individuals in or approaching this vulnerable age group need hope for “continuation of life” more than ever?

Furthermore, sufferers of all ages—including individuals in their 40s, 50s, 60s, 70s, or even higher—deserve to know that their age and longer illness duration do not diminish chances of recovery [10, 11].^2^ Ibrahim et al. [12] found that I-CBTE (integrated enhanced cognitive behavioural therapy) outcomes for AN inpatients were not predicted by age. In their words, “These findings give hope for people who have been chronically ill” [12, p. 9]. One of the inspiring patients involved in their study is quoted later in this article. Dr June Alexander is another remarkable individual who ‘turned the tables’ on her AN, after battling with it for decades (since the age of 11). She  emotively wrote in Treasure and Alexander [13]:

Easier said than done for someone with an eating disorder, but at age 55, I ticked all the boxes and crossed the line in regaining me. The years of struggle and hard work, tears and desperation, were over… Early intervention with family-based treatment is best by far, but no matter how long you have lived with an eating disorder—if you are 20, 30, 40, 50 or 60 or more—you can regain quality of life. You can be free. Yes, you can. [13, pp. vii–viii]

Through my own experiences as a patient and volunteer for the UK’s National Health Service (NHS), I have had the privilege of being able to meet ED sufferers in middle and late adulthood, who are living precious lives that they *want* to live. Gaudiani et al.'s [2] cut-off age would deprive people like them of the legitimate hope for a life worth living. Moreover, it implicitly sends a misleading message to ED professionals that some patients are ‘too old’ for recovery, and therefore not worth being offered recovery-based treatments. Implicit age bias is a huge problem in research also, and ideas like ‘terminal AN’ may reinforce this.

Furthermore, Gaudiani et al.'s [2] proposition of such a low cut-off age may have inadvertently given legitimacy to end-of-life decisions for patients who are not even close to reaching it. A sufferer in their early 20s, and even their clinicians, may question what difference a number makes when talking about young people in general. Specific numbers are often disregarded or forgotten. The idea that a young person can have a ‘terminal’ mental illness is dangerous in itself, regardless of the exact numbers conjectured. Anecdotally, I have recently heard (in radio interviews) and read (in social media posts) ED patients in their late teens and early 20s asking for end-of-life care through the NHS, usually because their treatment teams have seemingly given up on them.

## Criterion 3: “Previous participation in high-quality care” [2, p. 1]

Gaudiani et al. [2] proposed as criterion 3 in their original paper:

Prior persistent engagement in high-quality, multidisciplinary eating disorder care**.** Worldwide access to expert eating disorder care varies widely, as does the availability of access to expert inpatient, residential, and full day treatment programs for those with eating disorders. Thus, the definition of care identified here must remain somewhat broad. Before someone can decide they cannot recover, they must have participated in high-quality, expert care to the maximum extent that this is available. This provision should motivate policies that allow for transfers of patients out of designated ‘networks’ that lack expertise, with funding coverage provided at a centre of excellence. Ideally, at least some of this treatment will have been undertaken at a sufficiently high level of care to provide extensive structure and support, preferably to the point of full weight restoration at least once in the relatively recent past. Congruent with receipt of such care, qualified health care professionals on the team must support the patient in their decision to stop fighting. We acknowledge that many factors may impact patients’ ability to participate in such care, including lack of access to eating disorders expertise, limitations of the healthcare system, and a personal sense—often based on prior treatment experiences—that admission to certain care settings would cause more harm than good. [2, p. 11]

### “High-quality care” [2, p. 1]/“high-quality, multidisciplinary ED care” [2, p. 11]

The 3rd criterion states that previous experiences of “high-quality, multidisciplinary ED care” are necessary for a patient to be diagnosed with ‘terminal AN’. By implication, Gaudiani et al. [2] have excluded the vast majority of longstanding AN sufferers who have received inadequate ED care, or in many cases, no ED care at all. For those unfortunate individuals, the question of “engagement” cannot even apply. However, the 3rd criterion forgets about disadvantaged groups—including people who live where there is limited/no access to public health care due to socioeconomic deprivation, discriminated minority groups (e.g., ethnic minorities, LGBTQ + communities), and individuals with certain coexisting conditions (e.g.,  ASD, ADHD, learning difficulties).

Gaudiani et al. stated that a “definition of care identified here must remain somewhat broad” [2, p. 11]. However, life-or-death decisions should never be based on imprecise, vague definitions. If, as the authors acknowledge, precise definitions are impossible, then surely no set of criteria would be safely able to make a ‘terminal’ diagnosis? I address this issue later in the article, as it applies to all of the criteria.

As well as not adequately explaining the "definition of care identified here", Gaudiani et al. [2] have not indicated what type of it would qualify as being of a “high-quality”. Is it up to clinicians or patients to decide this? Just like the contrasting word “futile”, which the authors use in the 4th criterion, patients and clinicians rarely attribute the same meanings to the subjective rating of “high-quality”. For example, an uncompromising treatment approach may be considered abusive by one patient, but compassionate (at least in the long-term) by the clinician administering it. Another patient may appreciate the same ‘forceful’ approach because it allows them to not take responsibility for eating more (which can provoke huge amounts of anxiety and guilt)—‘I’m sorry AN, but I had no choice, so please don’t blame me.’ In this example, perhaps the meaning of “high-quality” would depend on the nature of the ‘compulsion’ used, assuming that 'compassionate compulsion' is possible.^3^ In any case, the use of compulsion must never be, or inadvertently become, abusive like the AN bully is.

Indeed, what qualifies as being “high-quality care” can be flexed by professionals, consciously or unconsciously, in order to protect themselves from criticism. Patients who do not respond to poor-quality care can be blamed for a lack of “previous participation” [2, p. 1] or “prior persistent engagement” [2, p. 11] with treatments that failed them (not vice versa). As stated in my previous article:

Apparent ‘treatment‑resistance’ is often caused when patients are, or have historically been, treated in a way that encourages resistance… Professionals must ask themselves whether the real problem is the delivery of treatment or the patient’s personal ‘resistance’ to it. [1, p. 2]

Gaudiani et al.'s [2] “broad” definition does not even distinguish between physical health care and mental health care. In the UK, most ED patients are discharged as soon as they are considered physically stable. At these times, they are often most vulnerable psychologically. Personally, I do not think that physical interventions should be considered forms of ‘care’ unless they are accompanied by meaningful, consistent, and sustained psychological care. Physical-only interventions are often iatrogenic due to the unnecessary use of coercion, and/or clinicians’ hyperfocus on calories and weight (to the exclusion of the deeply rooted psychological functions of ED behaviours). When clinicians fixate on calories and weight, they often counterproductively reinforce their patients’ surface obsessions.

Notably, most ED sufferers who identify with ‘terminal AN’ would (and have) self-diagnose(d) with it. They do not study the 3rd criterion, and objectively consider the quality of their past treatments, when they make negative assumptions (diagnoses) guided by cognitive biases (e.g., self-blaming, labelling, emotional reasoning, dichotomous thinking, selective abstraction)—biases which are exacerbated by the effects of malnutrition on the brain. Furthermore, these individuals rarely have opportunities to talk to professionals who can help them reframe their distorted cognitions. Nonetheless, the authors of ‘terminal AN’ seem to presume, in all of their publications to date [2, 3, 15], that every ED sufferer is also an ED patient with access to ED clinicians. This was manifested recently when Yager et al. [3] wrote:

Terms and concepts published in scientific journals can be misunderstood, misinterpreted, and misrepresented, and they do not come with trigger warnings. We fully appreciate the need for authors to use appropriate lay terms and sensitive language in the clinical context. At the same time, all clinicians who work with this population know that the capacity for the AN ‘voice’ to be triggered into ever-harsher, crueller demands and judgments is nearly infinite. If a casual comment on the street can do it, of course this discourse can too. Our intention is certainly not to cause distress, and individuals who are adversely affected by exposure to these terms might benefit from opportunities to further explore, clarify, and discuss their reactions to the issues raised by these concepts with their clinicians and others. [3, p. 2]

In reality, “triggered” ED sufferers who identify with ‘terminal AN’ would “discuss their reactions to the issues raised” with their AN voice. The authors rightly acknowledged in the same article that this voice has a “nearly infinite” capacity to make “ever-harsher, crueller demands and judgements” [3, p. 2]. It is  no wonder that many sufferers consequently assume death is the only possible option/outcome.

### “Previous participation” [2, p. 1]/“prior persistent engagement” [2, p. 11]

It is impossible for professionals to accurately, and retrospectively, calculate a patient’s previous “participation” or “engagement” levels. Professionals cannot confidently distinguish between patients who wish to engage with so-called “high-quality care”, and those who do not. Both stances usually co-occur and/or alternate in cycles of varying durations (from minutes/hours to months/years). Rankin et al. [14] demonstrated this when they found that patients receiving cognitive behavioural therapy (CBT) for anorexia nervosa (CBT-AN) experienced two concurrent identity-based “rhythms of motivation”, which the authors termed as “rapid cycling” and a “slow wave of change”. ‘Motivation’ and ‘engagement’ are of course linked—patients need to be motivated to engage, and they maintain this motivation by continuing to engage.

The subjects of ‘motivation’ and ‘engagement’ are strongly linked to assumptions about the purported ego-syntonicity of AN, a theory which suggests that when patients are not motivated to engage with “high-quality care”, they must value their AN as being harmonious with their total identity. I address these flawed assumptions later in the article.

## Criterion 4: “Clear and consistent determination by a patient who possesses decision-making capacity that additional treatment would be futile, knowing their actions will result in death.” [2, p. 1]

Gaudiani et al. [2] proposed as criterion 4 in their original paper:

Consistent, clear expression by an individual who possesses decision-making capacity that they understand further treatment to be futile, they choose to stop trying to prolong their lives, and they accept that death will be the natural outcome. Careful determination of decisional capacity is required in each case. An individual who wavers in their conviction or expresses different goals to different people is not yet ready to receive the appellation of terminal AN. [2, p. 11]

The 4th criterion arguably continues with the theme of ‘engagement’, by questioning whether a patient wishes to live (by engaging with treatment) or die (by disengaging with treatment and choosing to die instead). The safety of the ‘terminal AN’ hypothesis seems to rely most on the 4th criterion. In response to criticisms of their original paper [2], Yager et al. [15] asserted:

However, unless the patient otherwise meets all the criteria however, including that vital criterion 4 regarding their personal choice not to continue treatment while knowing death may follow, the term cannot be misused. [15, p. 8]

Unfortunately, “that vital criterion 4” is also the most confusing one in my opinion. Gaudiani et al. [2] have barely explained what is meant by its overlapping sub-themes:

### “Clear and consistent determination” [2, p. 1]/ “consistent, clear expression” [2, p. 11]

Gaudiani et al. [2] suggest that a patient’s wish to live or die can be reliably gauged by how “clear and consistent” their related declarations are. However, for the same reason that it is impossible to accurately measure ‘engagement’ and ‘participation’ levels, professionals cannot safely distinguish between patients who wish to live, or at least may wish to live in time, and those who want to die. Both stances usually co-occur and/or alternate in cycles of varying durations (from minutes/hours to months/years). Ambivalence about life/death can be very well camouflaged by the apparent “clear and consistent determination” to die. AN has an extremely vociferous voice that may silence any hope the patient has. Clinicians may hear the loud AN voice, but not the silent identity controlled by it. Sufferers can also be deafened by their own ED voice. In Yager et al.’s own words, “the capacity for the AN ‘voice’ to be triggered into ever-harsher, crueller demands and judgments is nearly infinite” [3, p. 2]. One of these cruel demands can be for the victim to refuse treatment and starve to death, even if doing so requires deceiving the people who care most.

To complicate matters further, ED sufferers are often unexpectedly articulate and persuasive, even when they are severely malnourished. The most overtly “clear and consistent determination” may hide deep, covert internal conflicts. Indeed, there are many ED sufferers who previously “clearly and consistently” refused life-saving treatments, but retrospectively accepted that those interventions were necessary/compassionate in the long-term [16, 17]. Anecdotally, some patients have even expressed gratitude to the clinicians who held the hope for them (for their *true* identities) when it would have been easier, or felt more empathic, to listen to the ED’s deceitful voice and label them as ‘treatment-resistant’ (or ‘terminal’ in extreme cases). As stated in my previous article:

Professionals who diagnose ‘terminal anorexia’ may inadvertently be agreeing with the sufferer’s AN ‘bully-friend’, which may tell them that because they cannot live without AN, they have no choice but to die from it. [1, p. 3]

Hence, it is imperative that clinicians fight compassionately for, but not coercively against, patients who are struggling to hold on to hope and protesting that they want to die. This does not justify using coercion by default. In my view, it is important to distinguish between emergency situations, such as when a patient is experiencing cardiac arrest, and urgent situations, such as when a patient is at risk of cardiac arrest but there is still time to actively listen to her/him, rather than automatically resort to force (which may ironically be more dangerous to patients who are physically weak, especially if they have osteoporosis). I genuinely believe that in the long-term, using patience and active listening—or at least more of it—is less time-consuming/expensive than using combative interventions to achieve short-term ‘tick-box’ outcomes. This is why I wrote in my previous article:

For example, a non‑combative approach might involve the clinician just being with the patient who refuses to eat, showing genuine and unconditional compassion and empathy, actively listening to the patient if they choose to talk, and holding the hope for them until they can realise that their life is worth living and their body is worth nourishing (even if, initially, they only agree to this for the clinician who is willing to sit patiently and unconditionally with them). Even when an approach like this does not [quickly] lead to the desired clinical outcome, it costs far less than forced and repeated medical interventions that have harmful outcomes. Unconditional patience costs far less than impatient reactions. [1, p. 2]

### “Decision-making capacity” [2, p. 1 and p. 11]

Despite masses of contentious research in all fields of psychiatry, there are still no agreed definitions of ‘decision-making capacity’, yet Gaudiani et al. [2] do not specify which of the many possible versions of it is appropriate for their 4th criterion. In my view, an AN-specific definition would first have to be agreed by ED professionals/researchers, and then accepted by professionals/researchers working in other mental health fields, as they would have to use the same term in different ways. If this is achievable, then the resulting definition would only be applicable to AN sufferers who have no coexisting conditions that may affect decision-making capacity. Gaudiani et al. themselves acknowledged in the 1st criterion that “patients with SEAN are likely to have other psychiatric conditions as well.” [2, p. 11]

Even if a reliable definition of ‘decision-making capacity’ can be agreed exclusively for the 4th criterion, whether or not a patient would technically meet the agreed threshold is not so important in my opinion. I believe that everyone with mental ill-health lacks capacity to some extent. Trying in vain to measure the precise extent can unnecessarily waste time and distract from more important, person-centred considerations. Often, an OCD patient’s obsessions are considered borderline delusional, but where the precise border technically lies between rational/overvalued and delusional ideas is usually not a key consideration when they are being treated by OCD professionals (at least in my own experience). On the other hand, capacity assessments are very frequently used for patients with psychosis, who have fully lost touch with reality and clearly surpassed any reasonable threshold.

Regardless of the precise definition used, even AN patients with overall ‘decision-making capacity’ are likely to experience significant confusion, especially while their brains are severely malnourished. Having overall ‘decision-making capacity’ and being very confused are not mutually exclusive states of mind. Thus, a confused patient’s declarations about wanting to die should never be readily accepted by clinicians, who may also be emotionally conflicted.^4^ Life-or-death decisions should not be made if there is any reasonable doubt, and it is unlikely that all involved parties (clinicians, patients, families, carers, and legal professionals if applicable) can assuredly make these judgements without any reasonable doubt. The enormously complex and deceitful nature of AN means that doubt held by professionals about a patient’s wish to live or die, *and* the patient’s own self-doubt about this (which I addressed in the previous subsection), can never be eliminated.

Gaudiani et al. have written that, “An individual who wavers in their conviction or expresses different goals to different people is not yet ready to receive the appellation of terminal AN” [2, p. 11]. This statement perplexes me. The longer a malnourished patient waits for the label, as they are “not yet ready” to receive it, the more their malnourished brain will be starved of decision-making capacity and hope. Surely, a patient who loses weight would also lose (or at least not gain) the cognitive ability to balance the pros and cons of living, as well as the interlinked emotional ability to hold hope for life? Malnutrition’s negative effect on the brain is a biological inevitability, just like its unavoidable effect on other organs, which the authors keenly stress in the 1st criterion (although the permanence of these effects is debatable). Therefore, how is it possible for a patient to *gain* decision-making capacity while they are malnourished? If a confused patient is only “ready to receive the appellation” *after* they have gained weight, then surely they would no longer meet the 1st criterion at that point? Indeed, the 1st criterion clearly states that a terminal diagnosis would only be warranted “should weight loss continue unabated.” [2, p. 11]

### “Additional treatment” [2, p. 1]/ “further treatment” [2, p.11]

Gaudiani et al. [2] have not explained what qualifies as being a ‘treatment’ for AN, let alone one that is evidence-based. Just like the related term “high-quality care”, determining what counts as a treatment is a totally subjective *and* evolving judgement. For example, past treatments based on operant conditioning techniques (which use rewards and punishments) [18] are now widely accepted to be unethical. In  England, the National Institute for Health and Care Excellence’s (NICE) currently recommends as treatment options for adult ED sufferers: CBT for eating disorders (CBT-ED), Maudsley Anorexia Nervosa Treatment for Adults (MANTRA), Specialist Supportive Clinical Management (SSCM), and Focal Psychodynamic Therapy (FPT) [19]. However, even in this advanced economy, most ED sufferers do not have access to the named treatments due to underfunding, staff shortages, bed shortages, inadequate training, and lack of mandatory monitoring. When evidence-based treatments are provided, this is often in a ‘watered down’ form.

Evidently, much more needs to be done to ensure that all longstanding ED sufferers have equitable access to the high-quality treatments that they deserve. Rather than recognise the injustice of this not happening, the 4th criterion makes individual patients take responsibility when professionals judge that “additional treatment would be futile” [2, p. 1] for them personally. Moreover, it discounts the potential of new or improved psychological (and possibly pharmacological^5^) treatments, which would be more constructive topics for future ED research than the defeatist idea of ‘terminal AN’. It would be a fatal tragedy to prematurely assume that all treatment options have been permanently exhausted for uniquely excluded individuals who are categorised as ‘terminal’. Demoralising assumptions like this are already being disproved by innovative and hopeful research. For example, in a recent study [12] of inpatient I-CBTE treatment, a very courageous individual is quoted as saying:

I had been suffering from a complex, co-morbid eating disorder for nearly 2 decades; I was locked away in psychiatric institutions as a ‘treatment resistant’, ‘revolving door patient’. The Oxford team provided a holistic, inclusive, ground-breaking and compassionate care with the I-CBTE model. I was able to work through all the issues and trauma of my long, terrible journey. For the first time ever, I reached a healthy weight. This helped me to think more clearly. I was encouraged to use my creativity, to imagine a new life. This was daunting, but exciting. On my discharge, I had support in the community, which helped me practice what I had learned in hospital and allowed my body to finish reaching its healthy weight. My illness was not really about weight, but learning to accept my body, whatever its weight, was essential. In the last 4 years, I have formed a completely new life for myself, which has nothing to do with eating disorders. This is the epitome of my recovery, the outcome of my I-CBTE treatment: I am successful, creative, needed. I will always be incredibly grateful to the multidisciplinary team in Oxford for their lifechanging care. [12, pp. 8–9]

Separately, Tchanturia et al. [20] have demonstrated the efficacy of integrated adjunct therapies based on remediation principles—namely cognitive remediation therapy (CRT) and cognitive remediation and emotion skills training (CREST). These treatments have shown promise in treating complex patients with coexisting  ASD. Motivational interviewing (MI) is another effective treatment adjunct [14], which appreciates the internal conflicts experienced by AN patients, who are often unrealistically expected in traditional treatments to take enormous leaps of faith into terrifying worlds without their ED ‘coping partners’. If possible, MI techniques should be used throughout treatment courses, not just at the start or before them—sustaining motivation is incredibly difficult for patients while they fight such a motivationally exhausting and hope-depriving illness. Additionally, I believe that MI should focus more on hope than willpower. AN sufferers already have plenty of willpower. However, without the hope for a meaningful life beyond their AN, this willpower is often channelled in self-destructive ways that are dictated by the AN (not by their true identities). Perhaps, there could be an AN-specific adaptation of motivational enhancement therapy in the form of a new ‘hope enhancement therapy’?

It is not just the names of treatments that matter. The 4th criterion has no way to measure the quality of therapeutic relationships, which I believe is more important for determining whether a treatment will be effective in individual cases. Due to its significance, I discuss what is required for ‘therapeutic alliances’ at the end of this article.

### “Futile” [2, p. 1 and p. 11]

Language matters enormously. To me, the impersonal word “futile” suggests failure. Patients labelled with ‘terminal AN’ have not failed treatment. In fact, it is far more likely that treatment has failed them. Unsurprisingly, patients who are given up on by professionals, and told that “additional treatment would be futile” [2, p. 1], are far more likely to give up on themselves also.

If a standard treatment (like CBT) does not produce the desired clinical outcomes for a patient, this does not mean a more flexible, holistic, and compassionate adaptation of it (like I-CBTE) would also be “futile” for the same patient. Additionally, genuinely high-quality and holistic treatments often do not ‘work’ in individual cases only because they are not given enough time to allow meaningful and sustainable gains. This is especially important for AN patients due to the time it takes to establish trustful therapeutic relationships, so cross-diagnostic comparisons of a therapy’s efficacy are unfair.

Furthermore, the measurable clinical outcomes desired by professionals often diverge from the goals of their patients. For some patients, recovery of quality and meaning of life are primary goals, not elimination of symptoms and total weight restoration (although the latter are more likely to also *become* primary goals if patients’ current wishes are respected). A patient has to be ready and determined to make the changes that they are being asked (or demanded) to make. Self-determination and patient autonomy are key, yet the 4th criterion dictates what ‘recovery’ should mean to individual patients, and therefore whether a treatment has been “futile” for them individually. Indeed, personal autonomy is a weak justification for ‘terminal AN’ in my opinion.

Importantly, a patient’s conception of ‘recovery’ may change over time. For every unique sufferer, recovery is an individual journey of discovery and acceptance—of their true/complete/far-reaching identities, their precious worth, and their valuable meaning/purpose in life. These journeys should not have time limits or set destinations. There are often unpredictable ‘turning points’, where sufferers who could not imagine life beyond AN discover something beautiful that fills the predicted emptiness, loneliness, and helplessness without it. This may be, for example, the birth of a child, a new friendship, an empathic and identity-appreciating therapist, or a role helping others with similar difficulties. Prematurely labelling these individuals with ‘terminal AN’ would deny them of the unpredictable and healing power of time. The author Hadley Freeman emotively captured this when she wrote in her book ‘Good Girls’ [23]:

“It seems such a silly, Hallmark movie end to this story: ‘sick for decades and then having kids made her all better!!!’ Children are not a cure for eating disorders, and mine didn’t cure me. It was time. I had outgrown that scratchy, self-made jumper of self-defeating self-destruction and I no longer believed that holding on to splinters of anorexia made me special. I truly wanted out, but I needed something to yank me out of it other than the needs of my own body and life, because I never cared about them. My children did the yanking. I had at last found something I cared about enough.” [23, p. 258] [Reprinted by permission of HarperCollins Publishers Ltd © (Hadley Freeman) (2023)]

### “Will result in death” [2, p. 1]/“death will be the natural outcome” [2, p. 11]

The 4th criterion’s prediction of death is dangerously ambiguous. When I first read the words “will result in death”, I naturally asked myself: If the effects of AN have reduced my life expectancy by X years, does that mean AN will be the cause of my death? What is the difference, if any, between ‘will result in my death’ and ‘will result in my earlier death’? How much ‘life left’ is necessary for me to fulfil the 4th criterion?

Confusingly to me, Gaudiani et al. [2] seem to have made inconsistent statements in their original and subsequent articles about ‘terminal AN’. These include:

As a result, consistent with literature on duration of life during hunger strikes resulting in death, a prognosis of less than 6 months can fairly be established when the patient acknowledges further treatment to be futile and stops engaging in active recovery work. A less than 6-month prognosis is congruent with current practice around determination of terminal diagnoses. [2, p. 11]

It is only when the palliative approach does not work, and death becomes imminent, that the patient meets the designation for terminal AN. [15, p. 8]

Our description of these patients’ last days and weeks as ‘terminal’ and meriting thoughtful end-of-life care is consistent with how the term is used in other end-stage terminal conditions. [3, p. 1]

The first quoted statement indicates that six months of life expectancy would satisfy the 4th criterion, so perhaps ‘terminal AN’ would be used to justify up to six months of palliative care? On the other hand, the second and third statements suggest that only a few days/weeks of life expectancy would satisfy the 4th criterion, so perhaps ‘terminal AN’ would be used to justify a few days/weeks of hospice care?

Arguably, the ‘terminal AN’ debate is fundamentally one of time. Thus, ambiguity about what is meant by “will result in death” is extremely perilous. Does it mean days, weeks, six months, more than six months, or an indefinite time period? Anecdotally, it is known that the label is already being used—formally and/or informally—for very young sufferers who potentially have decades of precious life ahead of them.

It is a heartbreaking reality that far too many AN sufferers die from the physical complications associated with this terrible illness (as well as from coexisting conditions that the 1st criterion overlooks). However, this unacceptable tragedy should never be accepted as an inevitability, even in the short-term. Making pessimistic prognoses may result in avoidable deaths inadvertently becoming more acceptable.^6^

## Ambiguous application of the criteria

The authors of the ‘terminal AN’ criteria have made various statements about how their criteria should be applied. These include:

As illustrated by our cases, no set of criteria will apply perfectly to every patient who identifies with having a terminal case of AN… Thus, the definition of care identified here [in the 3rd criterion] must remain somewhat broad. [2, p. 10 and p. 11]

This diagnostic subset of patients with terminal AN is not merely academic or theoretical in nature. In debating the specificity of the terminal designation, it is important to pause and consider how narrowing the definition further would play out in actual clinical practice. [15, p. 6]

However, unless the patient otherwise meets all the criteria however, including that vital criterion 4 regarding their personal choice not to continue treatment while knowing death may follow, the term cannot be misused… Given the vital importance of this term being applied strictly and rigorously, we wish to identify explicitly that terminal AN applies only to a rare subpopulation of all those with SEAN. [15, p. 8]

We clearly acknowledged that precise definition of the term remains to be developed and we invited the eating disorder and palliative care fields to systematically address these issues and develop consensus definitions and guidelines for these patients’ end-of-life care. [3, p. 2]

To me, these statements are very confusing. If the proposed criteria are “not merely academic or theoretical” because “no set of criteria will apply perfectly to every patient who identifies”, then how can they also be “applied strictly and rigorously”? How can a criterion that “must remain somewhat broad” also be “precise”? How can “precise” definitions be safely used in the real world, which is not “theoretical” like research hypotheses are?

If broad criteria are unsafe, and precise criteria are unachievable in the real world, then surely no set of criteria would be able to safely make a ‘terminal’ diagnosis of AN? Indeed, life-or-death clinical judgements should not be based on *any* criteria from my perspective. They should be profoundly considered on a case-by-case basis that appreciates each patient’s unique identity and set of circumstances. Applying ‘criteria’, however flexible they may be, diminishes and devalues the lived experiences of AN sufferers. Arguably, it is like subjecting them to an experimental hypothesis based on questionable assumptions.

When extraordinarily difficult and distressing clinical judgements are reluctantly made in heartbreaking individual cases, they do not also need to be formally labelled and advertised, in such a way that they can be easily replicated and applied to every AN sufferer who expresses the wish to die. Contrary to what Yager et al. hope, it is unlikely that their proposed criteria “applies only to a rare subpopulation of all those with SEAN” [15, p. 8]. Numerous sufferers I know have said that they would meet the proposed criteria, and many more identify with the term despite not strictly meeting its criteria. As stated earlier, sufferers do not study academic criteria when they make cognitively biased and emotional self-diagnoses, especially when they can identify with labels that promise to validate and end their suffering.

Even if Yager et al.'s latest requests for “precise definition of the term… [and] consensus definitions” [3, p. 2] are somehow achievable, training clinicians to safely apply them in the real world would be impossible.^7^ One error can have fatal consequences, not just on the labelled patient, but also on other sufferers if it sets a dangerous precedent that leads to domino effects.

## Confusing purpose of the criteria

In early 2022, I probably would have appreciated being given, or at least knowing that I could soon be given, a ‘terminal AN’ label. This would have given me permission to die the following year on my 30th birthday (a few days ago, at the time of writing). Therefore, I can understand why the concept was originally intended to alleviate the suffering experienced by AN patients who have overtly lost hope, as well as their loved ones, whom I particularly sympathise with. Indeed, Yager et al. [15] have explained that ‘terminal AN’ would be like a passport enabling them to get compassionate end-of-life care:

Defining these criteria for terminal AN is meant to cue practitioners about the potential need for a palliative approach or comprehensive end-of-life care plan. [15, p. 6]

However, compassion should not have to be prescribed, regardless of a patient’s stage in life. Formal ‘cues’ should not be necessary to trigger compassionate end-of-life care when it is required. It *goes without saying* that patients who need end-of-life care for any illness, and their loved ones, deserve to receive the *most* compassionate form of it possible (whether or not this care is formally titled as ‘end-of-life’, as there may not always be time to formalise it). When this does not happen, then surely the problem is a lack of compassion, not a lack of cues or labels? Surely, we should be arguing about this injustice instead?

Furthermore, in practical terms, I am unsure how labelling AN sufferers presumed to be in their “last days and weeks” [3, p. 1], or at a stage when their “death becomes imminent” [15, p. 8], would realistically benefit them. In fact, formal criteria might impose additional barriers on them, as they would now have to formally prove that they qualify for the compassionate end-of-life care promised by the appellation. Qualifying for this humane right should be an automatic process that does not require certificates.

Realistically, there would not even be enough time for every patient who identifies with ‘terminal AN’ to be safely assessed by one of the few (or zero) trained doctors in their country who would be qualified to formally diagnose it. Patients with truly ‘terminal’ illnesses do not have time to wait. They cannot delay their deaths, and they should not have to wait with uncertainty for the rest of their lives.

Notably, compassionate end-of-life care should not be conflated with compassionate care for patients who presently *feel* that death is the only option. Being categorised as ‘terminal’ would automatically remove any hope for life that these individuals have, which may be hidden/silenced by their AN. Further, it can implicitly instruct them to wait for their presumed deaths, rather than allow them to have the continued choice and hope of living. As stated earlier, personal autonomy is a weak justification for ‘terminal AN’ in my opinion.

## Impact of labelling, and how this relates to the therapeutic relationship

Gaudiani et al.'s [2] ‘terminal AN’ criteria were created for patients whom the authors consider infinitely ‘treatment-resistant’, to such an extent that their AN *specifically* is ‘terminal’. Labels such as ‘treatment-resistant’, ‘treatment-refractory’, and now ‘terminal’ have traditionally been linked to the theory of ‘ego-syntonicity’ (i.e., the idea that AN sufferers experience their mental illness as being harmonious with their total identity). Therefore, it is important to explore this assumption when considering the safety of the ‘terminal AN’ criteria, which arguably rely on it.

The Oxford Dictionary of Psychology [26] defines ‘ego-syntonic’ as:

Experienced as consistent or harmonious with the total personality. [26, p. 238]

On the opposite end of the spectrum, ‘ego-dystonic’ is defined as:

Experienced as self-repugnant, alien, discordant, or inconsistent with the total personality, as obsessions are generally experienced to be…. [26, p. 237]

Hence, the definition of ‘ego-syntonic’ can be used to explain the apparent lack of engagement and motivation of longstanding AN sufferers, in contrast to sufferers of ‘ego-dystonic’ mental illnesses like OCD. However, the lived reality for AN sufferers, who I do not believe can experience their distressing mental illness harmoniously, is a lot more complicated than the word ‘ego-syntonic’ implies. As stated in my previous article:

Importantly, ED sufferers do not *want* to make themselves more ill. The term ‘ego-syntonic’ is another label that is often used in an unhelpful and reductionist way. The reality for ED sufferers is usually a lot more complicated and less black-or-white (i.e., it is not just ‘ego-dystonic’ versus ‘ego-syntonic’) … Both inevitably intertwine and overlap, making it impossible to neatly separate them for the benefit of academic theories and treatment models. [1, p. 4]

I have often used the term ‘AN bully-friend’ to convey what it feels like when I experience these complicated conflicts. The two imperfect metaphors below demonstrate the ‘bullying’ (or ‘ego-dystonic’) side of AN more clearly, albeit in a simplistic and dichotomous way that I do not advocate being used in clinical practice unless patients can clearly identify. Characterisations like ’offender’, ‘victim’, and ‘rescuer’ are often inappropriate in the real world, so I have only used them to aid the imperfect metaphors.

Metaphor 1: AN sufferer as a hostage

A hostage has been forcibly isolated by their captor for many years. They counterintuitively fear being separated from their captor. They cannot imagine life without their captor. Their whole life *is* their captor (at the moment).

Metaphor 2: AN sufferer as an abused partner

An abused person has been coercively controlled by their partner for many years, and trained to feel helpless without their partner. They counterintuitively fear being separated from their partner. They cannot imagine life without their partner. Their whole life *is* their partner (at the moment).

In these two situations, the victim does not *truly* want/like the offender, though they may *feel* needing/deserving of the offender (at the moment). The victim does not want/like the negative self-beliefs and associated distressing emotions that make them feel needing/deserving of the offender.^8^ The victim does not ‘ego-syntonically’ choose to stay in the destructive (and possibly life-ending/terminal) relationship.

If a ‘rescuer’ (e.g., a therapist) makes the victim feel that they want, like, need, and/or deserve the offender, then the victim would inevitably feel more *unjustified* guilt, shame, and anxiety-driven dependency on the offender. Moreover, the victim would feel that they have no identity of their own, and are essentially owned by the offender. Hence, the victim needs the rescuer to tell them that they can *and* deserve to live without the offender; and that their genuine feelings of helplessness, loneliness, and emptiness without the offender would not be permanent or life-ending/terminal.

These two metaphors imperfectly exhibit the ‘ego-dystonic’ experience of AN, which I can *personally* relate to most, just like when I experience unwanted and intrusive obsessions caused by my OCD. This does *not* negate the ‘mixed up’ parts of me that often *feel* in the moment that AN provides relief from distress—something to fill the helplessness, loneliness, and emptiness when I have no other ‘coping companions’. Importantly, needing relief from distress is very different to wanting ego-syntonic pleasure. In my experience, it is not possible to feel pleasure while in the grips of an ED. They are mental illnesses, not lifestyle choices.

### Therapeutic alliances

Most patients who receive public health care experience multiple overlapping therapeutic relationships at the same time—with individual clinicians, local/specialist health services (often in the form of multidisciplinary teams), and even national health services. When clinicians and service providers use labels to define their patients, this significantly reflects on, and affects, the quality and dynamics of these relationships. Do they appreciate the patient as a unique individual with an identity extending far beyond a narrow label? Do they hold hope for the patient, and sincerely believe that she/he can recover a meaningful life worth living? Do they have the patience and time to allow the patient to discover their worth and meaning in life? Do they have the compassion and empathy required to provide person-centred treatment pathways, recognising that ‘recovery’ is an individual journey that cannot be mapped in advance using fixed signposts?

These are just some of the many questions that I hope the ‘terminal AN’ debate will inspire. Debating endlessly about the impersonal label itself, and trying to define its impersonal criteria, distracts from these more consequential and patient-oriented research questions.

Good therapeutic relationships (therapeutic *alliances*) require clinicians and service providers to appreciate their patients as unique individuals, with far-reaching identities that cannot be encapsulated by labels. Consequently, it is crucial that they strike an extremely sensitive and unique balance for every individual ED sufferer. As stated in my previous article:

In my view, there should be a balance between acknowledging how attached the sufferer may feel to their ED, especially if it genuinely helps them temporarily when they have nothing else to fall back on, and encouraging the sufferer to see their ED as something that should be ‘removed’ permanently because it is wholly evil. [1, p. 3]

Therapeutic *allies* help their patients realise that they can and deserve to live without their dishonest EDs. They help patients find honest alternatives that can shift the balance in disfavour of the EDs. Concurrently, they acknowledge that their patients may not yet be ready to accept these offers in full. When this happens, they do not label their patients as ‘treatment-resistant’ or ‘terminal’—the latter of which automatically deprives sufferers of the right, and the time, to discover the honest, and often beautiful, alternatives waiting to be found. As stated earlier, personal autonomy is a weak justification for ‘terminal AN’ in my opinion.

For several years, I have reflected on the main principles of care, or conditions, that are required for therapeutic alliances. These can also model to patients how they should treat themselves. It is impossible, and would be unhelpful, to create precise criteria. However, after deep contemplation, I recommend a general approach that involves humanity in the form of these key overlapping principles: Compassion, Hope, Empathy, Appreciation (of a patient’s true/complete/far-reaching identity), and Patience (which requires the subcomponents of active listening and curiosity). The ‘CHEAP’ approach is not expensive, and it does not require specialist training. There can be no excuses for poor-quality care, regardless of how this care is formally named and packaged.

Of course, the name of a treatment does matter significantly, largely for practical reasons such as funding, training, planning, monitoring, and research. Unfortunately, it is beyond the scope of this article to emphasise the urgent need for investment in the research, development, and provision of high-quality treatments for ED sufferers, regardless of their ages, illness durations, and (perceived) illness severities. These treatments may range from early interventions like ‘First Episode Rapid Early Intervention for Eating Disorders’ [27], to more intensive interventions like I-CBTE, which is effective for ED patients of all ages and illness durations. In general, I suggest that therapeutic approaches used in any therapy are more compassion-focused, emotion-focused, and humanistic, perhaps using identity-appreciating narrative therapy techniques.^9^ Ultimately, a tailored and person-centred integration of approaches is likely to be most effective in my view.

Supportive care, which is often named and packaged as ‘supportive psychotherapy’, does not need a formal name or prescription. Longstanding ED sufferers should have access to unconditional and ongoing supportive care, based on unconditional positive regard. It is unacceptable when this care is absent, and/or withdrawn due to negative regard encouraged by negative labels such as ‘treatment-resistant’ and ‘terminal’. Supportive care should consistently follow humanistic principles, such as those outlined in the ‘CHEAP’ approach, and it must not be terminated as soon as patients are considered physically stable—at these times, they are often most vulnerable and needing of being cared for. Indeed, for supportive care to be effective, the genuine loyalty of professionals/services must outweigh the perceived loyalty of the dishonest EDs, which longstanding ED sufferers are likely to have relied upon for many years.

## Conclusion

In my lived experience view, the whole concept of ‘terminal AN’ is harmful and unnecessary in the real world, regardless of what criteria might theoretically be agreed (if this is achievable, which I do not believe it is). The criteria proposed by Gaudiani et al. [2] are dangerously vague, reductionist, and possibly even dehumanising. They devalue the complexity and diversity of ED sufferers’ lived experiences. Moreover, to date, there has been a worrying lack of consideration of how the proposed criteria would be applied safely, and what realistic benefits doing this would confer to the labelled sufferers and their loved ones. I suggest that broader debates about patient autonomy, and the right to die, are not confused with ‘terminal AN’ considerations, as ‘terminal AN’ is a label that may inadvertently deny sufferers the right to live. Above all, we should be debating about how, as therapeutic *allies*, we can give ED sufferers the right to *truly* live—not just exist, survive, or wait to die.

## Data Availability

All supporting data are available within this article.

## Appendix

### Letter of concern to NHS England and NHS East of England, 19th September 2023

*While my article was in peer review, I requested to the journal editors that this letter, which I wrote after its submission, be included in the final publication (as an Appendix). This is because it links my article (especially the section on criterion 2) to the real world.*  

This letter of concern is written in response to a leaked NHS document (“Developing pathways for patients with longstanding eating disorder or severe and enduring eating disorder—L-ED/SEED”) that was exposed to the public in an online article [28] published by The Telegraph on 9th September 2023. The article reports that NHS East of England (NHS EoE), which is part of NHS England, produced guidance stating that longstanding eating disorder sufferers as young as 25 (or possibly even younger) may in some cases be given “the offer” of following a “palliative pathway".

In effect, NHS England is *offering*, at least to their patients in the East of England, a hope-depriving pathway that likely ends in death. The NHS (across all countries and regions of the UK) should instead be offering a hopeful pathway (or ‘recovery’ pathway) that allows eating disorder sufferers to discover their true/complete identities and precious meaning in life. When these individuals *feel* hopelessness, and in some heartbreaking cases express the wish to die, they deserve and need, more than ever, to be offered a hopeful pathway. Their *feelings*, which unlike death are not permanent, should be actively listened to and acknowledged, but not reinforced by the people who are supposed to hold the hope for them while they understandably struggle to carry it on their own.

Even if unintentionally, it is likely that the guidance, which ambiguously states that “pathways should not be age specific”, will influence treatment decisions for patients below the age of 25. The document recommends that clinicians treating patients as young as 18 are trained in end-of-life care, and it even provides a link to the e-learning programme “End-of-life Care for All”. Moreover, eating disorder patients below the age of 25, including children and adolescents, may now unjustifiably believe that NHS-sanctioned ‘palliative pathways’ offer future escape routes from the undeserved suffering that they *currently* experience (and *can* overcome).

The idea of offering a ‘palliative pathway’ to eating disorder sufferers of *all* ages is horrifying. Even more disturbingly, the guidance identifies a benefit as “reducing the costs associated with lengthy admissions to SEDU [specialist eating disorder units] or acute hospitals.” It is severe underfunding, and uncaring attempts to reduce costs, that lead to unnecessary considerations of palliative care in the first place.

Palliative care is not a solution to underfunding. The economic (and obviously humane) solution is to invest more resources into providing evidence-based and high-quality care/treatments (like I-CBTE), which includes the training of enough staff to deliver this care compassionately. Indeed, Baroness Parminter recently said in a parliamentary debate (on 29th June 2023) about how NHS eating disorder services are “failing patients” (due to treatment delays, bed shortages, and inadequate training—all of which are fixable problems caused by underfunding and mismanagement): I really am worried about the unnecessary deaths that are happening on this Government’s watch… I say to the Minister that, 6 years on, very little progress has been made. The people suffering from these vicious, cruel diseases deserve so much more. [25]

Rather than producing guidance on pathways to unnecessary deaths, NHS England’s Task and Finish Groups should instead spend their limited time writing guidance on high-quality care/treatments (like I-CBTE), so that patients finally receive the compassionate and holistic care that they so thoroughly deserve. All eating disorder sufferers can ‘recover’ according to their own definitions, which do not necessarily mean being ‘cured’, but do mean wanting to live, if they are given compassion *and* time. Suggestions to the contrary abdicate the NHS of its duty of care to them. As stated by Caroline Nokes MP after NHS EoE's guidance was revealed: … to put sufferers on a palliative pathway is just horrific… Sufferers need support at every level and at the right time, not to be written off in this way. [28]

More broadly, I am deeply concerned that  NHS EoE's ill-considered guidance has reinforced a growing (and misleading) international narrative that eating disorders, especially anorexia nervosa, can be ‘terminal’ illnesses that some sufferers can never ‘recover’ from. I recently expressed these concerns in an article published by the Journal of Eating Disorders in July 2023: Therefore, I was very disheartened when the label ‘terminal anorexia’ [and associated end-of-life considerations/language] was circulated by professionals. Research [and NHS EoE’s guidance] is not just read, seen, and heard about by the professionals who promote it. Vulnerable and conflicted eating disorder sufferers, and their families, can be victims of theoretical academic [and clinical/NHS EoE] discourse that has real-world, life-or-death implications. [1, p. 1]

I further state in a follow-up article that is under peer review: Disconcertingly, the proposition of such a low age threshold [25-years-old in the case of NHS EoE's guidance] may have inadvertently given legitimacy to end-of-life decisions for patients who are not even close to reaching it… Anecdotally, I have heard (in radio interviews) and read (in social media posts) eating disorder patients in their late teens and early 20s ask for end-of-life care, usually because their treatment teams have given up hope and/or refused additional treatments.

‘End-of-life’ pathways are often euphemised as ‘palliative’ pathways because they do not *necessarily* have to end in death—although disease progression is the most likely outcome according to most definitions, including that of the World Health Organisation [29], and most eating disorder sufferers like me naturally assume death when they hear the word ‘palliative’. So-called ‘palliative pathways’ ultimately lead patients down the same route—“I can offer you a comfortable route to death, but you can change your mind if you so choose.” The possibility of repeated U-turns inevitably makes sufferers more lost, confused, and internally conflicted.

Furthermore, ‘palliative pathways’ prevent patients from seeing alternative pathways that offer hope, and the opportunity for them to discover their true/complete identities and precious meaning in life. These journeys, which some may think of as ‘recovery’ pathways, do not have fixed timeframes and set destinations. They cannot be mapped in generic guidance that devalues individual sufferers.

NHS EoE's guidance suggests that at a given point in time, patients have a binary choice between ‘recovery’ and ‘harm-reduction’. However, these two approaches are not mutually exclusive. A ‘recovery’ pathway should unconditionally involve ‘harm-reduction’ strategies along the way if/when they are necessary, as well as compassionate guidance (not coercion) from professionals who hold the hope for their patients, even when their patients struggle to carry it on their own. Holding the hope means not giving up on the idea of ‘recovery’, a concept that should be defined by individual sufferers rather than by generic guidance.

## Abbreviations

ADHD: Attention deficit hyperactivity disorder
AN: Anorexia nervosa
ASD: Autism spectrum disorder
BN: Bulimia nervosa
CBT: Cognitive behavioural therapy
ED: Eating disorder
I-CBTE: Integrated enhanced cognitive behavioural therapy
MI: Motivational interviewing
NHS: National Health Service (UK)
NHS EoE: NHS East of England
OCD: Obsessive compulsive disorder
SEAN: Severe and enduring anorexia nervosa
SEED: Severe and enduring eating disorder
SMR: Standardised mortality rate

## Footnotes

1 I have often read that osteoporosis is the only irreversible physical condition caused by AN. However, even bone loss can be reversed in young adults according to rheumatologists that I have spoken to during treatment of my osteoporosis, which I was diagnosed with in my early 20s. Until bone development stops, I understand that renourishment and weight-bearing activities (in accordance with Wolff’s law) can restrengthen bones. There are also medical treatments, including bisphosphonates and testosterone therapy (as malnutrition markedly reduces active testosterone levels in males).

2 Notably, although the 2nd criterion assumes that older age automatically equates to longer duration of severe illness, this is not necessarily true (at least outside of planned research). ED sufferers older than 30 may have been diagnosed many years earlier, but only experienced ‘severe’ symptoms for a relatively short period of time. The terms ‘severe and enduring anorexia nervosa’ (SEAN) and ‘severe and enduring eating disorder’ (SEED) unhelpfully tie severity and chronicity together. This does not happen for other mental illnesses like dysthymia, which is a milder but more long-lasting form of depression.

3 *If* ‘compassionate compulsion’ is possible in rare individual cases, then it should not be defined by generic criteria. When professionals make clinical judgements on a case-by-case basis, they should ask themselves questions like: Is the ‘compulsion’ mild or severe? Is it physical (e.g., nasogastric tube-feeding under restraint), verbal (e.g., warnings of force-feeding), or written (e.g., contracts/prescriptions dictating what a patient must eat in order to stay out of hospital)? Is it accompanied by sustained psychological care during *and* after the intervention? These questions, and the hypothetical concept of ‘compassionate compulsion’, may be useful subjects  for future qualitative research.

4 Concurrently, professionals should always actively listen to, and take very seriously, a patient’s expressed wishes to refuse treatment and/or die. However, this does not require them to readily accept and/or agree with the patient’s declarations. Doing so may inadvertently collude with the AN voice, rather than be an empathic response to the patient’s true identity.

5 Discussion of novel pharmacological treatments is far beyond the scope of this paper. Nonetheless, it is worth briefly noting that psychedelics may be useful future treatment adjuncts. These include psilocybin, ketamine, lysergic acid diethylamide, and dimethyltryptamine [21]. A recent article by Peck et al. found that “psilocybin therapy is safe, tolerable and acceptable for female AN, which is a promising finding given physiological dangers and problems with treatment engagement.” [22, p. 1].

6 I urge ED researchers to adopt the same optimistic mentality that the COVID-19 vaccine pioneers did in 2020-2021. They disproved opposing scientists, who repeatedly insisted in 2020, using popular mainstream media (TV/radio) channels, that COVID-19 vaccines would take many years to develop at best, or never be attainable because attempts at producing vaccines for other coronaviruses had failed for decades. Broadcasting pessimism can negatively tip the balance for despairing ED sufferers who are contemplating death, just like it did for many dejected individuals who were encouraged to think that their lives had ended when COVID-19 lockdowns started.

7 Most doctors who diagnose EDs are poorly trained, even in advanced economies like the UK. Ayton and Ibrahim [24] found that the number of specialist ED consultants working in the UK was fewer than 100 in 2017, and non-specialist doctors usually had less than 2 h of ED training throughout their 10–16 years of medical education. The situation in the UK has not improved much since then. Baroness Kathryn Jane Parminter recently said in a parliamentary debate (on 29th June 2023) about how NHS eating disorder services are “failing patients”: *“However, I say to the Minister that, six years on, very little progress has been made. The people suffering from these vicious, cruel diseases deserve so much more."* [25]

8 For ED sufferers, feeling unworthy and needing/deserving of their illness does not mean that they value their perceived unworthiness and want their illness (which causes/perpetuates those feelings of worthlessness). However, it can be very confusing for sufferers to understand this, and make a distinction between desire and perceived need, while they are conflicted. Labels that suggest sufferers want to be ill may act as negative external reinforcers, thereby worsening the unjustified shame and guilt felt by some of them.

9 Perhaps, narrative therapy and creative therapy techniques can be integrated, so that patients may express themselves using whatever methods are most healing/cathartic for them personally. For example, this may be through letters, poetry, art, music, dance, drama, or any other creative mediums.
